# Simultaneous Detection of Key Bacterial Pathogens Related to Pneumonia and Meningitis Using Multiplex PCR Coupled With Mass Spectrometry

**DOI:** 10.3389/fcimb.2018.00107

**Published:** 2018-04-05

**Authors:** Chi Zhang, Leshan Xiu, Yan Xiao, Zhengde Xie, Lili Ren, Junping Peng

**Affiliations:** ^1^MOH Key Laboratory of Systems Biology of Pathogens, Institute of Pathogen Biology, Chinese Academy of Medical Sciences and Peking Union Medical College, Beijing, China; ^2^Christophe Mérieux Laboratory, Institute of Pathogen Biology, Chinese Academy of Medical Sciences and Peking Union Medical College, Beijing, China; ^3^Key Laboratory of Major Diseases in Children, Ministry of Education, National Key Discipline of Pediatrics, National Clinical Research Center for Respiratory Diseases, Beijing Key Laboratory of Pediatric Respiratory Infection Diseases, Virology Laboratory, Beijing Pediatric Research Institute, Beijing Children's Hospital, Capital Medical University, National Center for Children's Health, Beijing, China

**Keywords:** pneumonia, meningitis, bacterial infections, MALDI-TOF mass spectrometry, molecular diagnostic techniques

## Abstract

Pneumonia and meningitis continue to present an enormous public health burden and pose a major threat to young children. Among the causative organisms of pneumonia and meningitis, bacteria are the most common causes of serious disease and deaths. It is challenging to accurately and rapidly identify these agents. To solve this problem, we developed and validated a 12-plex PCR coupled with matrix-assisted laser desorption ionization-time of flight mass spectrometry (MALDI-TOF MS) method (bacterial pathogen-mass spectrometry, BP-MS) that can be used to simultaneously screen for 11 key bacterial pathogens related to pneumonia and meningitis. Forty-six nasopharyngeal swabs and 12 isolates were used to determine the specificity of the method. The results showed that, using the BP-MS method, we could accurately identify the expected bacteria without cross-reactivity with other pathogens. For the 11 target bacterial pathogens, the analytical sensitivity of the BP-MS method was as low as 10 copies/reaction. To further evaluate the clinical effectiveness of this method, 204 nasopharyngeal swabs from hospitalized children with suspected pneumonia were tested using this method. In total, 81.9% (167/204) of the samples were positive for at least one of the 11 target pathogens. Among the 167 bacteria-positive samples, the rate of multiple infections was 55.7% (93/167), and the most frequent combination was *Streptococcus pneumoniae* with *Haemophilus influenzae*, representing 46.2% (43/93) two-pathogen mixed infections. We used real-time PCR and nested PCR to confirm positive results, with identical results obtained for 81.4% (136/167) of the samples. The BP-MS method is a sensitive and specific molecular detection technique in a multiplex format and with high sample throughput. Therefore, it will be a powerful tool for pathogen screening and antibiotic selection at an early stage of disease.

## Introduction

Pneumonia and meningitis continue to be an enormous public health burden. According to the Global Burden of Disease 2015 study, more than 3.1 million deaths due to pneumonia and meningitis were estimated to have occurred in 2015. The two diseases together accounted for more than 15% of deaths in children under 5 years of age. Bacteria, viruses, and fungi can all cause pneumonia and meningitis, but bacteria are the leading cause of serious disease and deaths (GBD 2015 Mortality and Causes of Death Collaborators, 2016). For bacterial pathogens, the selection of appropriate antibiotics forms the basis of sound clinical management. However, this complex etiology and overlapping clinical presentations make it challenging to identify the causative organism.

Culture-based microbiological methods are routinely used to determine the responsible pathogen, but a long turn-around time and low sensitivity reduce their utility in making timely decisions (Prina et al., 2015). Even with an advanced automated bacteria identification system, nearly 20 h of processing time is needed (Bobenchik et al., 2014; Jacobs et al., 2017). Moreover, additional time and labor are necessary for pathogens that grow slowly, such as *Legionella pneumophila* and *Mycoplasma pneumoniae* (Atkinson et al., 2008; Mercante and Winchell, 2015). To avoid the drawbacks of culture-based approaches, molecular testing has been widely applied, providing an important complement to bacterial culture. Molecular detection is faster, more sensitive, and can be automated. In addition, several emerging technologies such as multiplex real-time PCR and microarray methods are available for clinical application (Buchan and Ledeboer, 2014).

MassARRAY System (Agena Bioscience, Inc., San Diego, CA, USA), a detection platform combining matrix-assisted laser desorption ionization-time of flight mass spectrometry (MALDI-TOF MS) with endpoint PCR, has been successfully applied in the field of microbial detection (Syrmis et al., 2011; Li et al., 2013; Peng et al., 2013a,b, 2014, 2016; Zhang et al., 2015). Taking advantage of the system's multiplex capability and high sample throughput, we developed and validated a bacterial pathogen-MS panel (BP-MS) to simultaneously screen 11 key bacterial pathogens associated with pneumonia and meningitis, including *Streptococcus pneumoniae, Haemophilus influenzae, Neisseria meningitidis, Klebsiella pneumoniae, Acinetobacter baumannii, Pseudomonas aeruginosa, Staphylococcus aureus, Moraxella catarrhalis, L. pneumophila, M. pneumoniae*, and *Bordetella pertussis*. To evaluate the effectiveness of the BP-MS method, we tested 204 clinical respiratory tract samples. Real-time PCR and nested PCR were used to confirm the results.

## Materials and methods

### Specimens

Using PCR and Sanger sequencing, 46 stock nasopharyngeal swabs were determined positive for target pathogens and used in the validation stage (data not shown). Isolates were also included in this validation stage. The 204 nasopharyngeal swabs used in the evaluation stage were collected from children hospitalized with suspected pneumonia in Beijing Children's Hospital in 2014. The swab samples were collected before antibiotic treatment.

### DNA extraction

All of the nasopharyngeal swabs were collected according to a standard protocol in general bacteria collection tubes with maintenance medium (Yocon, Beijing, China) and then stored at −80°C. For DNA extraction, samples were thawed at room temperature, 200 μl of maintenance medium was added, and the mixture was centrifuged at 5,000 × *g* for 10 min. The bacterial pellets were then resuspended in 150 μl of enzyme cocktail containing 30 U of lysostaphin, 6 mg of lysozyme, 37.5 U of mutanolysin, and 30 U of lyticase (Millipore Sigma, Darmstadt, Germany) in lysis buffer with working concentrations of 20 mM Tris-HCI (pH 8), 2 mM EDTA, and 1.2% Triton. To lyse the rigid cell walls of gram-positive bacteria, the resuspended bacteria were incubated at 37°C for 30 min. After incubation, 20 μl of proteinase K and 200 μl of buffer AL (QIAgen, Hilden, Germany) were added, and the mixture was incubated for 20 min at 56°C and then 15 min at 95°C. Finally, the DNA was purified using a QIAamp DNA Mini Kit (QIAgen, Hilden, Germany) according to the user's guide.

### Assay design

To design the assay, we used Assay Design 4.0 software (Agena Bioscience, Inc., San Diego, CA, USA) according to the user's manual. For each of the 11 pathogens, a target gene was chosen to design the detection assays. Human β-globin (HBB) was selected as an internal control, and BLASTn was used to check the specificity of each target gene. For each target gene, sequences in GenBank database were downloaded and aligned. Conserved regions were chosen to design the assays. In each assay, forward and reverse PCR primers were used to amplify the template and an extension primer was used for single base extension (SBE) reaction. To avoid interference in the mass spectra, a 10-base mass tag (ACGTTGGATG) was added to the 5′ end of each PCR primer. PCR and extension primers were synthesized by Tsingke Biological Technology (Beijing, China).

### Primary multiplex PCR and dephosphorylation reaction

Twelve pairs of PCR primers were pooled and then mixed with hot start PCR enzyme, PCR buffer, MgCl_2_ (Agena Bioscience, Inc.), uracil-DNA glycosylase (ShineGene Molecular Biotechnology, Shanghai, China), dNTP (dATP, dGTP, dCTP, and dUTP) (Promega, Madison, WI, USA), and 2 μl of DNA template for a 5-μl total volume. The multiplex PCR was performed in 384-well PCR plates using a ProFlex PCR system (Applied Biosciences, Foster City, CA, USA). The reaction conditions for the multiplex PCR were as follows: 45°C for 2 min; 94°C for 4 min; 95°C for 2 min; 45 cycles of 95°C for 30 s, 56.5°C for 30 s, and 72°C for 1 min; and 72°C for 5 min. After amplification, shrimp alkaline phosphatase (SAP) (Agena Bioscience, Inc.) was used to dephosphorylate excess dNTP at 37°C for 40 min, and then the SAP was inactivated at 85°C for 5 min.

### SBE reaction and MS

Following the dephosphorylation reaction, an SBE reaction was performed using the iPlex Pro SBE system (Agena Bioscience, Inc.) with a mixture of 12 extension primers according to the user's manual. During this process, the extension primers bound to the amplicons generated in the primary multiplex PCR and incorporated one terminator nucleotide. After exchanging salts with resin, the SBE reaction products were transferred to a 384 silica array preloaded with matrix (Agena Bioscience, Inc.) and then analyzed by mass spectrometry. If an extension primer incorporated a terminator nucleotide, a peak representing the extended extension primer appeared at the expected mass. The peak height representing the original extension primer was reduced. Based on the mass spectrum, the template could be identified. The detailed procedure with reagent concentrations and reaction conditions can be found in previous reports (Zhang et al., 2015; Xiu et al., 2017).

### Real-time PCR and nested PCR methods used as confirmatory tests

Real-time PCR was performed to confirm positive results from the BP-MS method. For *S. pneumoniae, H. influenzae, S. aureus, M. catarrhalis, K. pneumoniae, A. baumannii*, and *P. aeruginosa*, multiplex real-time PCR was used (Gadsby et al., 2015). For *N. meningitidis, L. pneumophila, M. pneumoniae*, and *B. pertussis*, singleplex real-time PCR was used according to previously published methods (Corless et al., 2001; Welti et al., 2003; Tatti et al., 2011). Each sample was tested in duplicate and regarded as positive if both cycle threshold (CT) values were <40. Nested PCR methods were used to resolve disagreements between results of the BP-MS and real-time PCR methods. The nested PCR was designed in-house for this study and was based on published conventional PCR assays. The primers and probes used in the confirmatory tests described above are listed in Tables S2, S3 in the Supplementary Material.

### Statistical analysis

Differences in the concordance rates of results for multiple-infection samples and single-infection samples were χ^2^-tested. A *p*-value <0.05 was considered statistically significant.

### Ethics approval

The study was performed in accordance with the recommendations of national ethics regulations and approved by the Institutional Review Board of the Institute of Pathogen Biology. All participants provided written informed consent in accordance with the Declaration of Helsinki. For children, written informed consent was obtained from parents or guardians.

## Results

### BP-MS method

In this study, we developed a 12-plex method to simultaneously detect 11 key bacterial pathogens associated with pneumonia and meningitis, using HBB as a nucleic acid extraction control. On the basis of previously published studies, we chose well-characterized and highly sensitive and specific genes as targets. *In silico* analysis showed that the BP-MS method can be used to specifically detect target pathogens, including most strains within a species. The final target genes and assays are listed in Table S1 in the Supplementary Material.

### Validation of specificity and sensitivity

**Figure 1:**
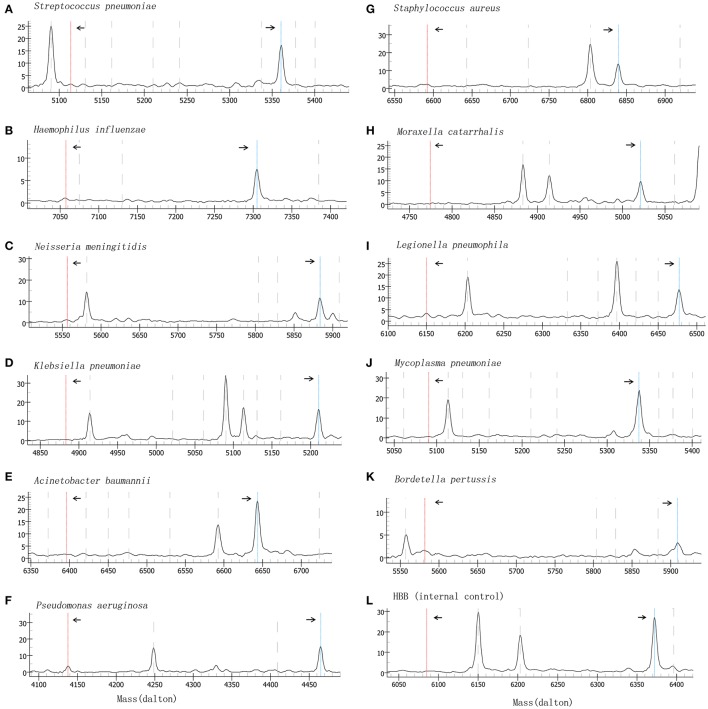
Mass spectrum of sensitivity evaluation by using 10-fold diluted plasmids. Figures **(A–L)** represents the mass spectra of each target by analyzing plasmids at 10 copies/reaction. The left arrow indicates the consumed unextended primer and the right arrow indicates the extended primer. The x-axis represents the mass of extension primer and the y-axis represents the intensity.

Specificity of the method was validated by testing 58 confirmed clinical samples and isolates (Table 1). The results showed that the BP-MS method can be used to accurately identify the expected bacteria without cross-reactivity with non-target pathogens (Figures S1, S2 in the Supplementary Material). Plasmids containing the target genes of each pathogen were 10-fold diluted and used to evaluate the analytical sensitivity of the BP-MS method. Based on the results, the BP-MS method can be used to determine the concentration of corresponding plasmids with 10 copies per reaction (Figure 1).

**Table 1:** Sequencing confirmed nasopharyngeal swabs and isolates used for validating specificity.

**Target bacteria**	**No. of confirmed swabs**	**No. of isolates**	**Total**
*Streptococcus pneumoniae*	7	0	7
*Haemophilus influenzae*	6	1	7
*Neisseria meningitidis*	2	2	4
*Klebsiella pneumoniae*	3	2	5
*Acinetobacter baumannii*	3	1	4
*Pseudomonas aeruginosa*	3	1	4
*Staphylococcus aureus*	6	0	6
*Moraxella catarrhalis*	5	1	6
*Legionella pneumophila*	0	4	4
*Mycoplasma pneumoniae*	7	0	7
*Bordetella pertussis*	4	0	4
Total	46	12	58

### Evaluation of the BP-MS method performance with clinical samples

A total of 204 nasal and throat swabs were collected to evaluate the performance of the BP-MS method. Each of the 11 target pathogens was detected, with an overall detection rate of 81.9% (167/204). Among all the 167 pathogen-positive samples, the rate of infection with multiple pathogens was 55.7% (93/167). Two and three pathogens were detected in 47 and 29 samples, respectively, whereas four or five pathogens were detected in 17 samples.

Samples determined pathogen-positive by the BP-MS method were re-tested first by real-time PCR. Using a combination of multiplex and singleplex real-time PCRs, 46.1% (77/167) of the positive samples showed results concordant with those from the BP-MS method. Compared with the BP-MS method, 56.8% (42/74) of the single-infection samples showed concordant results, but for multiple infection samples, the concordance rate was only 37.6% (35/93). These concordance rates differed significantly (*p* = 0.014 by χ^2^-test). The discordant samples were further confirmed by nested PCR followed by sequencing of PCR products. Combining results of the real-time and nested PCRs, the BP-MS method and confirmatory tests showed 81.4% (136/167) concordance, with verification rates for each target pathogen ranging from 50% (*L. pneumophila*) to 100% (*B. pertussis*) (Table 2). For single-infection samples, the overall verification rate was 81.1% (60/74), whereas this rate was 81.7% (76/93) for multiple-infection samples. The difference between these two rates was not statistically significant (*p* = 0.92 by χ^2^-test). The most frequent combination in multiple-pathogen infections was *S. pneumoniae* with *H. influenzae*, which accounted for 46.2% (43/93) of mixed infections. All combinations of multiple infections that were verified by confirmatory tests in this study are listed in Table 3. Thirty-one BP-MS-positive samples were not verified by either real-time PCR or nested PCR. Of these, 14 were single-infection samples and 17 were multiple-infection samples. All single-infection samples tested negative by both confirmatory tests, and, for 13 of 17 multiple-infection samples, one of the targets was not confirmed. For single-infection samples, *S. pneumoniae* was the pathogen most commonly unconfirmed (*n* = 9) whereas for multiple-infection samples, *K. pneumoniae* was the pathogen most commonly unconfirmed (*n* = 7). Details of the 31 discordant samples are shown in Table 4.

**Table 2:** Results of confirmatory tests of positive samples by the BP-MS method.

**Target bacteria**	**No. (%) of positive samples by BP-MS**	**No. of samples confirmed by real-time PCR/No. of positive samples by BP-MS**	**Verification rate (%) of real-time PCR**	**No. of discordant samples confirmed by nested PCR**	**No. of overall confirmed samples**	**Overall verification rate (%)**
*Streptococcus pneumoniae*	124 (60.8)	72/124	58.1	40	112	90.3
*Haemophilus influenzae*	56 (27.5)	52/56	92.9	2	54	96.4
*Neisseria meningitidis*	1 (0.5)	0/1	0	1	1	100
*Klebsiella pneumoniae*	25 (12.3)	1/25	4	15	16	64
*Acinetobacter baumannii*	23 (11.3)	11/23	47.8	7	18	78.3
*Pseudomonas aeruginosa*	13 (6.4)	9/13	69.2	2	11	84.6
*Staphylococcus aureus*	25 (12.3)	17/25	68	7	24	96
*Moraxella catarrhalis*	27 (13.2)	24/27	88.9	2	26	96.3
*Legionella pneumophila*	2 (1)	1/2	50	0	1	50
*Mycoplasma pneumoniae*	25 (12.3)	21/25	84	1	22	88
*Bordetella pertussis*	3 (1.5)	3/3	100	0	3	100
Total^a^	167(81.9)	77/167	46.1	59	136	81.4

a *The total number of clinical samples is 204*.

**Table 3:** Multiple infections verified by confirmatory tests.

**Infection type^a^**	**No**.	**Infection type**	**No**.	**Infection type**	**No**.
**Dual infections (*n* = 40)**		**Triple infections (*n* = 24)**		**Quadruple infections (*n* = 11)**	
SP, HI	15	SP, HI, MC	4	SP, HI, KP, AB	2
SP, PA	5	SP, HI, AB	4	SP, MP, HI, SA	1
SP, MP	3	SP, HI, SA	3	HI, MC, KP, PA	1
SP, SA	3	SP, SA, MC	3	SP, MC, KP, AB	1
SP, MC	3	SP, MP, HI	3	SP, MP, MC, AB	1
SP, AB	2	SA, MC, KP	2	SP, MP, HI, KP	1
KP, AB	2	KP, PA, AB	1	SP, HI, MC, KP	1
MP, HI	2	SP, HI, PA	1	SP, HI, MC, NM	1
SA, KP	2	MP, HI, KP	1	SP, HI, SA, MC	1
SA, MC	1	SP, MP, MC	1	SP, BP, SA, AB	1
HI, KP	1	SP, MP, PA	1	Quintuple infections (n = 1)	
HI, MC	1			SP, MP, HI, SA, MC	1

a *SP, Streptococcus pneumoniae; LP, Legionella pneumophila; BP, Bordetella pertussis; MP, Mycoplasma pneumoniae; HI, Haemophilus influenzae; SA, Staphylococcus aureus; MC, Moraxella catarrhalis; KP, Klebsiella pneumoniae; PA, Pseudomonas aeruginosa; AB, Acinetobacter baumannii; NM, Neisseria meningitides*.

**Table 4:** Samples with discordant results by the BP-MS method and the confirmatory tests.

**Sample ID**	**BP-MS results^a^**	**Confirmatory tests results**
S1	PA	Negative
S2	SP	Negative
S3	SP	Negative
S4	AB	Negative
S5	KP	Negative
S6	SP	Negative
S7	SP	Negative
S8	LP	Negative
S9	SP	Negative
S10	SP	Negative
S11	SP	Negative
S12	SP	Negative
S13	SP	Negative
S14	HI	Negative
S15	SP, SA, KP, AB	SP, SA, AB
S16	SP, HI, AB	SP, HI
S17	SP, AB	SP
S18	HI, MC, KP, PA	HI, MC
S19	SP, MP	SP
S20	SP, HI, SA	SP, SA
S21	SP, BP, HI	BP, HI
S22	SP, MC, KP, AB	SP
S23	SP, MP, HI, SA	SP, MP, HI
S24	MP, PA	PA
S25	SA, KP, AB	SA
S26	SP, LP, KP	SP, LP
S27	KP, AB	AB
S28	SP, KP	KP
S29	SP, MP	SP
S30	SP, KP	Negative
S31	SP, HI, KP, AB	SP, HI, AB

a *SP, Streptococcus pneumoniae; LP, Legionella pneumophila; BP, Bordetella pertussis; MP, Mycoplasma pneumoniae; HI, Haemophilus influenzae; SA, Staphylococcus aureus; MC, Moraxella catarrhalis; KP, Klebsiella pneumoniae; PA, Pseudomonas aeruginosa; AB, Acinetobacter baumannii; NM, Neisseria meningitidis*.

## Discussion

Pneumonia and meningitis can be caused by various bacterial pathogens; therefore, identifying the causative organism in a clinical case is challenging. Real-time PCR is a method frequently used for pathogen detection, but the limited optical channels restrict the number of targets that can be detected simultaneously. An optimized real-time PCR assay can be used to detect three to four targets in one reaction, which means that multiple reactions are needed to cover the spectrum of potential pathogens (Edin et al., 2015; Gadsby et al., 2015). Some sample-to-result platforms, such as the FilmArray (Biofire, Salt Lake City, UT, USA) system, provide another solution. The FilmArray system integrates sample preparation, amplification, detection, and analysis in one device, and the results can be obtained in 1 h, with 5 min of hands-on time. However, each FilmArray analyzer can process only one specimen per run, resulting in a high cost per specimen (Poritz et al., 2011; Babady, 2013).

One advantage of the BP-MS method established in this study is the comprehensive coverage of targets. Up to 11 pathogens can be detected simultaneously in one reaction, which is beneficial in respiratory illnesses with many possible etiologies and saves a substantial amount of labor. The high sample throughput of this method is another advantage. Using a 384-well PCR plate, as many as 380 clinical specimens can be analyzed within a single experiment, reducing the cost to <$4 per sample (not including nucleic acid extraction). Therefore, when many samples need to be tested, the BP-MS method is more feasible and economical than a FilmArray system.

The 11 target pathogens selected for use in the BP-MS method are the etiologic organisms most frequently detected in association with bacterial respiratory diseases. For community acquired pneumonia (CAP), *S. pneumoniae* is most often the cause, and other common bacterial causes include *H. influenzae* and *M. catarrhalis* (Musher and Thorner, 2014). *M. pneumoniae* and *L. pneumophila* are atypical CAP etiologies (Arnold et al., 2007). In contrast to bacteria associated with CAP, the dominant etiologic agents of hospital-acquired bacterial pneumonia are *S. aureus, P. aeruginosa, K. pneumoniae*, and *A. baumannii*, always showing less susceptibility to antimicrobials (Jones, 2010). In addition, *N. meningitidis* and *B. pertussis* are common causes of meningitis and pertussis. We have also designed assays for *Chlamydia pneumoniae*, which is an important atypical pathogen responsible for CAP. As isolates and positive clinical samples of *C. pneumoniae* are not available in this study, we have not evaluated its performance, so it is not included in the current panel.

Based on the sensitivity test, the limit of detection (LOD) of the BP-MS method is 10 gene copies/reaction. In this study, 31 samples positive for at least one of the target pathogens using the BP-MS method were not confirmed positive by either real-time PCR or nested PCR. We speculate that the different designs of the BP-MS method and confirmatory tests accounted for such discrepancies. Apart from methodological factors, the lower LOD with the BP-MS method may be attributable to the five additional cycles of primary multiplex PCR and dissimilar target selection (*H. influenzae* and *S. aureus*, Table S2). For target pathogens with low rates of concordance, such as *K. pneumoniae*, this disagreement may be attributable to differences in sensitivities of the BP-MS method and confirmatory tests.

The composition of the airway microbiota is driven by different ecological factors and is highly heterogeneous (Huffnagle et al., 2017). Some commensal bacteria such as *Streptococcus mitis, Streptococcus oralis*, and *Haemophilus haemolyticus* are closely related to pathogenic bacteria, which complicates accurate identification in certain individuals. Because of resource constraints, isolates of commensal bacteria were not included in the validation stage. The increase in detection with the BP-MS method relative for that with real-time PCR and nested PCR may be the results of cross-reactions with related species. Although we chose species-specific genes as targets to avoid cross-reactions, this possibility cannot be ruled out.

For CAP cases, multiple types of specimens should be used for microbial diagnosis (Johansson et al., 2010). A positive result from blood or pleural fluid (e.g., for meningitis, cerebrospinal fluid) samples by molecular test is of great value for pathogen identification. However, some substances present in blood, body fluids, and sputum may affect polymerase activity and inhibit amplification (Burd, 2010). Because of this, in the next stage of evaluation, various specimen types will be included.

The BP-MS method is a qualitative test, and its high sensitivity may allow asymptomatic carriage to be incorrectly interpreted as an etiological diagnosis. *S. pneumoniae* is the most commonly pathogenic bacteria carried in the nasopharyngeal cavity. The prevalence of *S. pneumoniae* carriage differs greatly between countries, ranging from 8.6 to 72%, and is higher in developing countries (Marchisio et al., 2002; Huang et al., 2004; Hill et al., 2006; Neves et al., 2013). Two recent studies indicate that the colonization rate of *S. pneumoniae* among healthy children in China is 16.6 to 26.6% (Hu et al., 2016; Pan et al., 2016), which means that for some cases of pneumonia, *S. pneumoniae* may not be the causative agent. Therefore, *S. pneumoniae* in a respiratory tract sample should be interpreted clinically, as is the case with other colonizing microorganisms such as *H. influenzae, S. aureus, M. catarrhalis*, and *N. meningitidis*. In this respect, a quantitative molecular test is more helpful in distinguishing etiological infection from asymptomatic carriage (Gadsby et al., 2015), whereas the BP-MS method can provide guidance on target selection for quantitative tests.

Using conventional and real-time PCR techniques, microbial etiologies can be identified in 65 to 86% of CAP cases (Thomson and Harris, 2011). For bacterial etiologies, detection rates are reported to be 58% by Johansson et al. (2010) and 44.7% by Jones (2010). Still nearly half of pneumonia cases, in which no causative agents can be detected (Mandell et al., 2007). In the present study, using the BP-MS method, the rate of bacterial pathogen detection was 81.9% (167/204), which indicates that this new method can assist determining the causative pathogens in cases with unknown etiologies.

Mixed infections have been reported by different groups, with the rate of bacterial coinfection varying from 13% to approximately 33%; the most common combination is *S. pneumoniae* and *H. influenzae* (de Roux et al., 2006; Cilloniz et al., 2011b; Musher et al., 2017). Bacterial coinfection is associated with severe disease and high mortality rates, so timely discovery of mixed etiologies is critical for antimicrobial treatment and clinical outcomes (Cilloniz et al., 2011a,b; Kumagai et al., 2015). In our study, *S. pneumoniae* and *H. influenzae* were the most frequently identified coinfection bacteria, but multiple infections were found in 45.6% (93/204) of samples, which is higher than the percentage determined in other studies. In particular, we noticed that, when using real-time PCR alone, the concordance rate of results for samples with multiple infections was lower than that for single-infection samples. This finding suggests that the BP-MS method performs well in clarifying complicated polymicrobial etiologies.

In summary, with the advantages of its multiplex format and high sample throughput, the BP-MS method is a powerful tool that can be used for pathogen screening and may provide valuable information for antibiotic selection in early stages of disease.

## Author contributions

JP, LR, and ZX conceived the experiments and analyzed the results. CZ conducted the experiments, analyzed the results, and wrote the manuscript. LX conducted the experiments and analyzed the results. YX collected specimens. All authors reviewed the manuscript.

### Conflict of interest statement

The authors declare that the research was conducted in the absence of any commercial or financial relationships that could be construed as a potential conflict of interest.

## References

[B1] Arnold F. W. Summersgill J. T. Lajoie A. S. Peyrani P. Marrie T. J. Rossi P.. (2007). A worldwide perspective of atypical pathogens in community-acquired pneumonia. Am. J. Respir. Crit. Care Med. 175, 1086–1093. 10.1164/rccm.200603-350OC 17332485

[B2] Atkinson T. P. Balish M. F. Waites K. B. (2008). Epidemiology, clinical manifestations, pathogenesis and laboratory detection of *Mycoplasma pneumoniae* infections. FEMS Microbiol. Rev. 32, 956–973. 10.1111/j.1574-6976.2008.00129.x 18754792

[B3] Babady N. E. (2013). The FilmArray(R) respiratory panel: an automated, broadly multiplexed molecular test for the rapid and accurate detection of respiratory pathogens. Expert Rev. Mol. Diagn. 13, 779–788. 10.1586/14737159.2013.848794 24151847 PMC7103684

[B4] Bobenchik A. M. Hindler J. A. Giltner C. L. Saeki S. Humphries R. M. (2014). Performance of Vitek 2 for antimicrobial susceptibility testing of *Staphylococcus* spp. and *Enterococcus* spp. J. Clin. Microbiol. 52, 392–397. 10.1128/JCM.02432-13 24478467 PMC3911353

[B5] Buchan B. W. Ledeboer N. A. (2014). Emerging technologies for the clinical microbiology laboratory. Clin. Microbiol. Rev. 27, 783–822. 10.1128/CMR.00003-14 25278575 PMC4187641

[B6] Burd E. M. (2010). Validation of laboratory-developed molecular assays for infectious diseases. Clin. Microbiol. Rev. 23, 550–576. 10.1128/CMR.00074-09 20610823 PMC2901657

[B7] Cilloniz C. Ewig S. Ferrer M. Polverino E. Gabarrus A. Puig de la Bellacasa J.. (2011a). Community-acquired polymicrobial pneumonia in the intensive care unit: aetiology and prognosis. Crit Care 15:R209. 10.1186/cc1044 21914220 PMC3334753

[B8] Cilloniz C. Ewig S. Polverino E. Marcos M. A. Esquinas C. Gabarrus A.. (2011b). Microbial aetiology of community-acquired pneumonia and its relation to severity. Thorax 66, 340–346. 10.1136/thx.2010.143982 21257985

[B9] Corless C. E. Guiver M. Borrow R. Edwards-Jones V. Fox A. J. Kaczmarski E. B. (2001). Simultaneous detection of *Neisseria meningitidis, Haemophilus influenzae*, and *Streptococcus pneumoniae* in suspected cases of meningitis and septicemia using real-time PCR. J. Clin. Microbiol. 39, 1553–1558. 10.1128/JCM.39.4.1553-1558.2001 11283086 PMC87969

[B10] de Roux A. Ewig S. Garcia E. Marcos M. A. Mensa J. Lode H.. (2006). Mixed community-acquired pneumonia in hospitalised patients. Eur. Respir. J. 27, 795–800. 10.1183/09031936.06.00058605 16585087

[B11] Edin A. Granholm S. Koskiniemi S. Allard A. Sjostedt A. Johansson A. (2015). Development and laboratory evaluation of a real-time PCR assay for detecting viruses and bacteria of relevance for community-acquired pneumonia. J. Mol. Diagn. 17, 315–324. 10.1016/j.jmoldx.2015.01.005 25772704 PMC7185852

[B12] Gadsby N. J. McHugh M. P. Russell C. D. Mark H. Conway Morris A. Laurenson I. F.. (2015). Development of two real-time multiplex PCR assays for the detection and quantification of eight key bacterial pathogens in lower respiratory tract infections. Clin. Microbiol. Infect. 21, 788 e781-788 e713. 10.1016/j.cmi.2015.05.004 25980353 PMC4509705

[B13] GBD 2015 Mortality and Causes of Death Collaborators (2016). Global, regional, and national life expectancy, all-cause mortality, and cause-specific mortality for 249 causes of death, 1980-2015: a systematic analysis for the Global Burden of Disease Study 2015. Lancet 388, 1459–1544. 10.1016/S0140-673631012-1 27733281 PMC5388903

[B14] Hill P. C. Akisanya A. Sankareh K. Cheung Y. B. Saaka M. Lahai G.. (2006). Nasopharyngeal carriage of *Streptococcus pneumoniae* in Gambian villagers. Clin. Infect. Dis. 43, 673–679. 10.1086/506941 16912937

[B15] Hu J. Sun X. Huang Z. Wagner A. L. Carlson B. Yang J.. (2016). *Streptococcus pneumoniae* and *Haemophilus influenzae* type b carriage in Chinese children aged 12-18 months in Shanghai, China: a cross-sectional study. BMC Infect. Dis. 16:149. 10.1186/s12879-016-1485-3 27080523 PMC4831093

[B16] Huang S. S. Finkelstein J. A. Rifas-Shiman S. L. Kleinman K. Platt R. (2004). Community-level predictors of pneumococcal carriage and resistance in young children. Am. J. Epidemiol. 159, 645–654. 10.1093/aje/kwh088 15033642

[B17] Huffnagle G. B. Dickson R. P. Lukacs N. W. (2017). The respiratory tract microbiome and lung inflammation: a two-way street. Mucosal Immunol. 10, 299–306. 10.1038/mi.2016.108 27966551 PMC5765541

[B18] Jacobs M. R. Mazzulli T. Hazen K. C. Good C. E. Abdelhamed A. M. Lo P.. (2017). Multicenter clinical evaluation of BacT/ALERT VIRTUO blood culture system. J. Clin. Microbiol. 55, 2413–2421. 10.1128/JCM.00307-17 28539343 PMC5527419

[B19] Johansson N. Kalin M. Tiveljung-Lindell A. Giske C. G. Hedlund J. (2010). Etiology of community-acquired pneumonia: increased microbiological yield with new diagnostic methods. Clin. Infect. Dis. 50, 202–209. 10.1086/648678 20014950 PMC7107844

[B20] Jones R. N. (2010). Microbial etiologies of hospital-acquired bacterial pneumonia and ventilator-associated bacterial pneumonia. Clin. Infect. Dis. 51(Suppl. 1), S81–S87. 10.1086/653053 20597676

[B21] Kumagai S. Ishida T. Tachibana H. Ito Y. Ito A. Hashimoto T. (2015). Impact of bacterial coinfection on clinical outcomes in pneumococcal pneumonia. Eur. J. Clin. Microbiol. Infect. Dis. 34, 1839–1847. 10.1007/s10096-015-2421-y 26059041

[B22] Li K. Guo J. Zhao R. Xue Y. Chen L. Yang J.. (2013). Prevalence of 10 human polyomaviruses in fecal samples from children with acute gastroenteritis: a case-control study. J. Clin. Microbiol. 51, 3107–3109. 10.1128/JCM.01324-13 23824769 PMC3754648

[B23] Mandell L. A. Wunderink R. G. Anzueto A. Bartlett J. G. Campbell G. D. Dean N. C.. (2007). Infectious diseases society of America/American thoracic society consensus guidelines on the management of community-acquired pneumonia in adults. Clin. Infect. Dis. 44(Suppl. 2), S27–S72. 10.1086/511159 17278083 PMC7107997

[B24] Marchisio P. Esposito S. Schito G. C. Marchese A. Cavagna R. Principi N.. (2002). Nasopharyngeal carriage of *Streptococcus pneu*moniae in healthy children: implications for the use of heptavalent pneumococcal conjugate vaccine. Emerging Infect. Dis. 8, 479–484. 10.3201/eid0805.010235 11996682 PMC2732490

[B25] Mercante J. W. Winchell J. M. (2015). Current and emerging Legionella diagnostics for laboratory and outbreak investigations. Clin. Microbiol. Rev. 28, 95–133. 10.1128/CMR.00029-14 25567224 PMC4284297

[B26] Musher D. M. Abers M. S. Bartlett J. G. (2017). Evolving understanding of the causes of pneumonia in adults, with special attention to the role of pneumococcus. Clin. Infect. Dis. 65, 1736–1744. 10.1093/cid/cix549 29028977 PMC7108120

[B27] Musher D. M. Thorner A. R. (2014). Community-acquired pneumonia. N. Engl. J. Med. 371, 1619–1628. 10.1056/NEJMra1312885 25337751

[B28] Neves F. P. Pinto T. C. Correa M. A. dos Anjos Barreto R. de Souza Gouveia Moreira L. Rodrigues H. G.. (2013). Nasopharyngeal carriage, serotype distribution and antimicrobial resistance of *Streptococcus pneumoniae* among children from Brazil before the introduction of the 10-valent conjugate vaccine. BMC Infect. Dis. 13:318. 10.1186/1471-2334-13-318 23849314 PMC3718621

[B29] Pan H. Cui B. Huang Y. Yang J. Ba-Thein W. (2016). Nasal carriage of common bacterial pathogens among healthy kindergarten children in Chaoshan region, southern China: a cross-sectional study. BMC Pediatr. 16:161. 10.1186/s12887-016-0703-x 27741941 PMC5064895

[B30] Peng J. Gao L. Guo J. Wang T. Wang L. Yao Q.. (2013a). Type-specific detection of 30 oncogenic human papillomaviruses by genotyping both E6 and L1 genes. J. Clin. Microbiol. 51, 402–408. 10.1128/JCM.01170-12 23152557 PMC3553860

[B31] Peng J. Li K. Zhang C. Jin Q. (2016). MW polyomavirus and STL polyomavirus present in tonsillar tissues from children with chronic tonsillar disease. Clin. Microbiol. Infect. 22, 97 e91-97 e93. 10.1016/j.cmi.2015.08.028 26363407

[B32] Peng J. Wang T. Zhu H. Guo J. Li K. Yao Q.. (2014). Multiplex PCR/mass spectrometry screening of biological carcinogenic agents in human mammary tumors. J. Clin. Virol. 61, 255–259. 10.1016/j.jcv.2014.07.010 25088618

[B33] Peng J. Yang F. Xiong Z. Guo J. Du J. Hu Y.. (2013b). Sensitive and rapid detection of viruses associated with hand foot and mouth disease using multiplexed MALDI-TOF analysis. J. Clin. Virol. 56, 170–174. 10.1016/j.jcv.2012.10.020 23194776

[B34] Poritz M. A. Blaschke A. J. Byington C. L. Meyers L. Nilsson K. Jones D. E.. (2011). FilmArray, an automated nested multiplex PCR system for multi-pathogen detection: development and application to respiratory tract infection. PLoS ONE 6:e26047. 10.1371/annotation/468cfdcd-184c-42f7-a1d0-3b72a2f6a558 22039434 PMC3198457

[B35] Prina E. Ranzani O. T. Torres A. (2015). Community-acquired pneumonia. Lancet 386, 1097–1108. 10.1016/S0140-6736(15)60733-4 26277247 PMC7173092

[B36] Syrmis M. W. Moser R. J. Whiley D. M. Vaska V. Coombs G. W. Nissen M. D.. (2011). Comparison of a multiplexed MassARRAY system with real-time allele-specific PCR technology for genotyping of methicillin-resistant *Staphylococcus aureus*. Clin. Microbiol. Infect. 17, 1804–1810. 10.1111/j.1469-0691.2011.03521.x 21595795

[B37] Tatti K. M. Sparks K. N. Boney K. O. Tondella M. L. (2011). Novel multitarget real-time PCR assay for rapid detection of *Bordetella* species in clinical specimens. J. Clin. Microbiol. 49, 4059–4066. 10.1128/JCM.00601-11 21940464 PMC3232951

[B38] Thomson A. Harris M. (2011). Community-acquired pneumonia in children: what's new? Thorax 66, 927–928. 10.1136/thoraxjnl-2011-200671 21933948

[B39] Welti M. Jaton K. Altwegg M. Sahli R. Wenger A. Bille J. (2003). Development of a multiplex real-time quantitative PCR assay to detect *Chlamydia pneumoniae, Legionella pneumophila* and *Mycoplasma pneumoniae* in respiratory tract secretions. Diagn. Microbiol. Infect. Dis. 45, 85–95. 10.1016/S0732-889300484-4 12614979

[B40] Xiu L. Zhang C. Wu Z. Peng J. (2017). Establishment and application of a universal coronavirus screening method using MALDI-TOF mass spectrometry. Front. Microbiol. 8:1510. 10.3389/fmicb.2017.01510 28848521 PMC5552709

[B41] Zhang C. Xiao Y. Du J. Ren L. Wang J. Peng J.. (2015). Application of multiplex PCR coupled with matrix-assisted laser desorption ionization-time of flight analysis for simultaneous detection of 21 common respiratory viruses. J. Clin. Microbiol. 53, 2549–2554. 10.1128/JCM.00943-15 26019198 PMC4508422

