# Supplementary material for: Simultaneous Detection of Key Bacterial Pathogens Related to Pneumonia and Meningitis Using Multiplex PCR Coupled With Mass Spectrometry
Source: Front Cell Infect Microbiol. 2018 Apr 5;8:107. doi: 10.3389/fcimb.2018.00107 (PMC5895723; doi:10.3389/fcimb.2018.00107)
Supplement: Supplementary file 1 [file Table1.PDF]

Supplementary Material

Simultaneous Detection of Key Bacterial Pathogens Related to Pneumonia and Meningitis by Using Multiplexed PCR Coupled with Mass Spectrometry

Chi Zhang[1]†, Leshan Xiu[1]†, Yan Xiao[1, 2], Zhengde Xie[3]*, Lili Ren[1, 2]*, Junping Peng[1]*

* these authors are corresponding authors.

† these authors contributed equally to this work.

Correspondence:

Junping Peng, pengjp@hotmail.com

Lili Ren, renliliipb@163.com

Zhengde Xie, zhengdexie@bch.com.cn

Supplementary information

Table S1 Target gene and sequences of amplification primers and extension primers used in the BP-MS method

Table S2 Primers and probes of real-time PCR used in this study

Table S3 Primers of nested PCR used in this study

Fig S1 Evaluation the specificity of the assay of *S. pneumoniae*, *H. influenzae*, *N. meningitidis, K. pneumoniae, A. baumannii,* and *P. aeruginosa*.

Fig S2 Evaluation the specificity of the assay of *S. aureus*, *M. catarrhalis*, *L. pneumophila*, *M. pneumoniae, B. pertussis,* and HBB.

Table S1 | Target gene and sequences of amplification primers and extension primers used in the BP-MS method

Target pathogen	Target Gene	Forward Primer	Reverse Primer	Extension Primer	Reference
Streptococcus pneumoniae	*lytA*	ACGTTGGATGTGGCAC TTGGTACTACTTTG	ACGTTGGATGAGTACC AGTTGCCGTCIGTG	GCGCTGTGCTTCCTCCA	(Carvalho Mda et al., 2007)
Haemophilus influenzae	*hpd*	ACGTTGGATGGTTCTT TTACCAACGIAGTG	ACGTTGGATGAAATATG CCGATGGTGTTGG	GTTAATAAAGAAGAATCCAAACC	(Wang et al., 2011)
Neisseria meningitidis	*ctrA*	ACGTTGGATGGGGAG AACACAAGAAATCGG	ACGTTGGATGATCTTGC AAACCGCCCATAC	GGCTTCAGAAAGCGATAA	(Corless et al., 2001)
Klebsiella pneumoniae	*gltA*	ACGTTGGATGCGCTGA AAATGCTGGAAGAG	ACGTTGGATGGGCGGA AAGAGTCATTCTTG	GCACGACGAACAAATT	(Gadsby et al., 2015)
Acinetobacter baumannii	*bla*$_{\text{OXA-51-like}}$	ACGTTGGATGCACGCT TCACTTCCTTAGAC	ACGTTGGATGATGAAA GCTTCCGRTATTC	CTATCAAGATTTAGCTCGTCG	(Turton et al., 2006)
Pseudomonas aeruginosa	*gyrB*	ACGTTGGATGGCATGT ACATCGGCGACAC	ACGTTGGATGTCGTCG ATGGAGTTATCCAC	CCACCACCTCGAAC	(Gadsby et al., 2015)
Staphylococcus aureus	*femB*	ACGTTGGATGTGACCT TGATGAATATGTG	ACGTTGGATGCGCCAT CATTTGYTCACGAC	CCTTACTTTTCTTTGTCATTCA	(Klotz et al., 2003)
Moraxella catarrhalis	*copB*	ACGTTGGATGTTATGC TAGACCCCCAGATG	ACGTTGGATGATTGGTT GCCCCAATGCCTG	CTGCCCCTTTTTGTAC	(Greiner et al., 2003)
Legionella pneumophila	*mip*	ACGTTGGATGATTCTT CCCCAAATCGGCAC	ACGTTGGATGACCGAT GCCACATCYTTAGC	GAGGTCATTAGCTACAGACA	(Hayden et al., 2001)
Mycoplasma pneumoniae	P1 adhesion gene	ACGTTGGATGTTAAGC CCTGAGAGGACAAG	ACGTTGGATGCAGTGA GCTTATTGGACCAG	CATCCCTGTCAAGCTCA	(Gullsby et al., 2008)
Bordetella pertussis	pertussis toxin subunit S1	ACGTTGGATGACGAG CACGACACGTGGTT	ACGTTGGATGATCCGG CTCTTGAGCGCATA	GGGATATGGCAAAGCCAA	(Tatti et al., 2011)
Internal control	HBB	ACGTTGGATGACTGTG CTTGACCTGGGAAC	ACGTTGGATGAAAGCA GCACTTGACTAGAG	ACCCCAAGTGAGACATTTTA	

References

Carvalho Mda, G., Tondella, M.L., Mccaustland, K., Weidlich, L., Mcgee, L., Mayer, L.W., Steigerwalt, A., Whaley, M., Facklam, R.R., Fields, B., Carlone, G., Ades, E.W., Dagan, R., and Sampson, J.S. (2007). Evaluation and improvement of real-time PCR assays targeting lytA, ply, and psaA genes for detection of pneumococcal DNA. *J Clin Microbiol* 45**,** 2460-2466.

Corless, C.E., Guiver, M., Borrow, R., Edwards-Jones, V., Fox, A.J., and Kaczmarski, E.B. (2001). Simultaneous detection of Neisseria meningitidis, Haemophilus influenzae, and Streptococcus pneumoniae in suspected cases of meningitis and septicemia using real-time PCR. *J Clin Microbiol* 39**,** 1553-1558.

Gadsby, N.J., Mchugh, M.P., Russell, C.D., Mark, H., Conway Morris, A., Laurenson, I.F., Hill, A.T., and Templeton, K.E. (2015). Development of two real-time multiplex PCR assays for the detection and quantification of eight key bacterial pathogens in lower respiratory tract infections. *Clin Microbiol Infect* 21**,** 788 e781-788 e713.

Greiner, O., Day, P.J., Altwegg, M., and Nadal, D. (2003). Quantitative detection of Moraxella catarrhalis in nasopharyngeal secretions by real-time PCR. *J Clin Microbiol* 41**,** 1386-1390.

Gullsby, K., Storm, M., and Bondeson, K. (2008). Simultaneous detection of Chlamydophila pneumoniae and Mycoplasma pneumoniae by use of molecular beacons in a duplex real-time PCR. *J Clin Microbiol* 46**,** 727-731.

Hayden, R.T., Uhl, J.R., Qian, X., Hopkins, M.K., Aubry, M.C., Limper, A.H., Lloyd, R.V., and Cockerill, F.R. (2001). Direct detection of Legionella species from bronchoalveolar lavage and open lung biopsy specimens: comparison of LightCycler PCR, in situ hybridization, direct fluorescence antigen detection, and culture. *J Clin Microbiol* 39**,** 2618-2626.

Klotz, M., Opper, S., Heeg, K., and Zimmermann, S. (2003). Detection of Staphylococcus aureus enterotoxins A to D by real-time fluorescence PCR assay. *J Clin Microbiol* 41**,** 4683-4687.

Tatti, K.M., Sparks, K.N., Boney, K.O., and Tondella, M.L. (2011). Novel multitarget real-time PCR assay for rapid detection of Bordetella species in clinical specimens. *J Clin Microbiol* 49**,** 4059-4066.

Turton, J.F., Woodford, N., Glover, J., Yarde, S., Kaufmann, M.E., and Pitt, T.L. (2006). Identification of Acinetobacter baumannii by detection of the blaOXA-51-like carbapenemase gene intrinsic to this species. *J Clin Microbiol* 44**,** 2974-2976.

Wang, X., Mair, R., Hatcher, C., Theodore, M.J., Edmond, K., Wu, H.M., Harcourt, B.H., Carvalho Mda, G., Pimenta, F., Nymadawa, P., Altantsetseg, D., Kirsch, M., Satola, S.W., Cohn, A., Messonnier, N.E., and Mayer, L.W. (2011). Detection of bacterial pathogens in Mongolia meningitis surveillance with a new real-time PCR assay to detect Haemophilus influenzae. *Int J Med Microbiol* 301**,** 303-309.
