# Supplementary material for: Simultaneous Detection of Key Bacterial Pathogens Related to Pneumonia and Meningitis Using Multiplex PCR Coupled With Mass Spectrometry
Source: Front Cell Infect Microbiol. 2018 Apr 5;8:107. doi: 10.3389/fcimb.2018.00107 (PMC5895723; doi:10.3389/fcimb.2018.00107)
Supplement: Supplementary file 2 [file Table2.PDF]

Supplementary Material

Simultaneous Detection of Key Bacterial Pathogens Related to Pneumonia and Meningitis by Using Multiplexed PCR Coupled with Mass Spectrometry

Chi Zhang[1]†, Leshan Xiu[1]†, Yan Xiao[1, 2], Zhengde Xie[3]*, Lili Ren[1, 2]*, Junping Peng[1]*

* these authors are corresponding authors.

† these authors contributed equally to this work.

Correspondence:

Junping Peng, pengjp@hotmail.com

Lili Ren, renliliipb@163.com

Zhengde Xie, zhengdexie@bch.com.cn

Supplementary information

Table S1 Target gene and sequences of amplification primers and extension primers used in the BP-MS method

Table S2 Primers and probes of real-time PCR used in this study

Table S3 Primers of nested PCR used in this study

Fig S1 Evaluation the specificity of the assay of *S. pneumoniae*, *H. influenzae*, *N. meningitidis*, *K. pneumoniae*, *A. baumannii*, and *P. aeruginosa*.

Fig S2 Evaluation the specificity of the assay of *S. aureus*, *M. catarrhalis*, *L. pneumophila*, *M. pneumoniae*, *B. pertussis*, and HBB.

Table S2 | Primers and probes of real-time PCR used in this study

Target pathogen	Forward Primer	Reverse Primer	Probe	Target Gene	Reference
Streptococcus pneumoniae	ACGCAATCTAGCAGATGAAGCA	TCGTGCGTTTTAATTCCAGCT	TGCCGAAAACGCTTGATACAGGGAG	*lytA*	(Gadsby et al., 2015)
*Haemophilus influenzae**	ATGGCGGGAACATCAATGA	ACGCATAGGAGGGAAATGGTT	CGGTAATTGGGATCCAT	*fucK*	
*Staphylococcus aureus**	AGCATCCTAAAAAAGGTGTAGAGA	CTTCAATTTTMTTTGCATTTTCTACCA	TTTTCGTAAATGCACTTGCTTCAGGACCA	*nuc*	
Moraxella catarrhalis	CGTGTTGACCGTTTTGACTTT	CATAGATTAGGTTACCGCTGACG	ACCGACATCAACCCAAGCTTTGG	*copB*	
Klebsiella pneumoniae	AGGCCGAATATGACGAAT	GGTGATCTGCTCATGAA	ACTACCGTCACCCGCCACA	*gltA*	
Pseudomonas aeruginosa	CCTGACCATCCGTCGCCACAAC	CGCAGCAGGATGCCGACGCC	CCGTGGTGGTAGACCTGTTCCCAGACC	*gyrB*	
Acinetobacter baumannii	TTTAGCTCGTCGTATTGGACT	CCTCTTGCTGAGGAGTAATTTT	TGGCAATGCAGATATCGGTACCCA	$bla_{\text{OXA-51-like}}$	
Neisseria meningitidis	GCTGCGGTAGGTGGTTCAA	TTGTCGCGGATTTGCAACTA	CATTGCCACGTGTCAGCTGCACAT	*ctrA*	(Corless et al., 2001)
Legionella pneumophila	AAAGGCATGCAAGACGCTATG	TGTTAAGAACGTCTTTCATTTGCTG	TGGCGCTCAATTGGCTTTAACCGA	*mip*	(Welti et al., 2003)
Mycoplasma pneumoniae	CCAACCAAACAACAACGTTCA	ACCTTGACTGGAGGC CGTTA	TCAATCCGAATAACGGTGACTTCTTACCACTG	P1 adhension gene	
Bordetella pertussis-IS*481**	CAAGGCCGAACGCTTCAT	GAGTTCTGGTAGGTGTGAGCGTAA	CAGTCGGCCTTGCGTGAGTGGG	IS*481*	(Tatti et al., 2011)
Bordetella pertussis-ptxS1	CGCCAGCTCGTACTTC	GATACGGCCGGCATT	AATACGTCGACACTTATGGCGA	*ptxS1*	

* For these pathogens, target genes selected for the BP-MS method were different from that of real-time PCR.

References

Corless, C.E., Guiver, M., Borrow, R., Edwards-Jones, V., Fox, A.J., and Kaczmarski, E.B. (2001). Simultaneous detection of Neisseria meningitidis, Haemophilus influenzae, and Streptococcus pneumoniae in suspected cases of meningitis and septicemia using real-time PCR. *J Clin Microbiol* 39(4)**,** 1553-1558. doi: 10.1128/JCM.39.4.1553-1558.2001.

Gadsby, N.J., McHugh, M.P., Russell, C.D., Mark, H., Conway Morris, A., Laurenson, I.F., et al. (2015). Development of two real-time multiplex PCR assays for the detection and quantification of eight key bacterial pathogens in lower respiratory tract infections. *Clin Microbiol Infect*. doi: 10.1016/j.cmi.2015.05.004.

Tatti, K.M., Sparks, K.N., Boney, K.O., and Tondella, M.L. (2011). Novel multitarget real-time PCR assay for rapid detection of Bordetella species in clinical specimens. *J Clin Microbiol* 49(12)**,** 4059-4066. doi: 10.1128/JCM.00601-11.

Welti, M., Jaton, K., Altwegg, M., Sahli, R., Wenger, A., and Bille, J. (2003). Development of a multiplex real-time quantitative PCR assay to detect Chlamydia pneumoniae, Legionella pneumophila and Mycoplasma pneumoniae in respiratory tract secretions. *Diagn Microbiol Infect Dis* 45(2)**,** 85-95.
