# Supplementary material for: Simultaneous Detection of Key Bacterial Pathogens Related to Pneumonia and Meningitis Using Multiplex PCR Coupled With Mass Spectrometry
Source: Front Cell Infect Microbiol. 2018 Apr 5;8:107. doi: 10.3389/fcimb.2018.00107 (PMC5895723; doi:10.3389/fcimb.2018.00107)
Supplement: Supplementary file 3 [file Table3.PDF]

Supplementary Material

Simultaneous Detection of Key Bacterial Pathogens Related to Pneumonia and Meningitis by Using Multiplexed PCR Coupled with Mass Spectrometry

Chi Zhang[1]†, Leshan Xiu[1]†, Yan Xiao[1, 2], Zhengde Xie[3]*, Lili Ren[1, 2]*, Junping Peng[1]*

* these authors are corresponding authors.

† these authors contributed equally to this work.

Correspondence:

Junping Peng, pengjp@hotmail.com

Lili Ren, renliliipb@163.com

Zhengde Xie, zhengdexie@bch.com.cn

Supplementary information

Table S1 Target gene and sequences of amplification primers and extension primers used in the BP-MS method

Table S2 Primers and probes of real-time PCR used in this study

Table S3 Primers of nested PCR used in this study

Fig S1 Evaluation the specificity of the assay of *S. pneumoniae*, *H. influenzae*, *N. meningitidis, K. pneumoniae, A. baumannii,* and *P. aeruginosa*.

Fig S2 Evaluation the specificity of the assay of *S. aureus*, *M. catarrhalis*, *L. pneumophila*, *M. pneumoniae, B. pertussis,* and HBB.

Table S3 | Primers of nested PCR used in this study

Target Pathogen	Direction	Target gene	1st Primers	2nd Primers [a]	Reference
Streptococcus pneumoniae	Forward<br>Reverse	*lytA*	GGCTACTGGTACGTACATTC<br>AATCAAGCCATCTGGCTCTA	ATCCAAAAGACAAGTTTGAGA<br>CTGGATAAAGGCATTTGATAC	(Messmer et al., 1997)
Haemophilus influenzae	Forward<br>Reverse	*hpd*	ATTGCTCACCGTGGTGCTAG<br>ACACCATCGGCATATTTAACCAC	TGGTCGTTTAGTGGTTATTCACG<br>CCCATTTGTGGAAGTAATTCCGT	This study
Staphylococcus aureus	Forward<br>Reverse	*nuc*	GTTTCGAAAGGGCAATACGCA<br>ACATAAGCAACTTTAGCCAAGCC	AGCGATTGATGGTGATACGGT<br>TCGTTTACCATTTTTCCATCAGCA	This study
Moraxella catarrhalis	Forward<br>Reverse	16S rRNA	CCCATAAGCCCTGACGTTAC<br>CTACGCATTTCACCGCTACAC	ND	(Hendolin et al., 1997)
Klebsiella pneumoniae	Forward<br>Reverse	*gltA*	GATGTGCTAAAAGGCACGCT<br>CATGCGCAGGAAGTTGCC	GTAGTCTTGGTTCAAAAGGCGT<br>TCGTTGCGCGGATAAACAAA	This study
Pseudomonas aeruginosa	Forward<br>Reverse	*gyrB*	GGAGAATCCCAACGAAGCCA<br>ATGAAGTGCTCGGTCAGCTC	AGGGCAAGATCCTCAACGTC<br>GCCTGGTCGTCCTTGATGTA	This study
Acinetobacter baumannii	Forward<br>Reverse	$bla_{\text{OXA-51-like}}$	AACGAAGCACACACTACGGG<br>TCGAACAGAGCTAGGTATTCCTTT	CTCGTGCTTCGACCGAGTAT<br>AGCTTGTAAGCAAACTGTGCC	This study
Neisseria meningitidis	Forward<br>Reverse	*ctrA*	GCTGCGGTAGGTGGTTCAA<br>TTGTCGCGGATTTGCAACTA	ND	(Tzanakaki et al., 2005)
Legionella pneumophila	Forward<br>Reverse	*mip*	GGGGCTTGCAATGTCAACAG<br>AATAGGTCCGCCAACGCTAC	GCAACCGATGCCACATCATT<br>GGCAATACAACAACGCCTGG	This study
Mycoplasma pneumoniae	Forward<br>Reverse	P1 adhension gene	CCATGGGTGATACCGCTACC<br>ACGCGAAGACTTGCAACTCT	AACCTTGTCGGGAAGAGCTG<br>TCAAGCTCACATGCCACTGT	This study
Bordetella pertussis	Forward<br>Reverse	IS481	GACTTCGTCTTCGTGGCCAT<br>GTACAGCGCGCCCGATGCCT	CGCGTGGCCTTCACCGACAT<br>GGGCGGTAAGGTCGGGTAAA	(Farrell et al., 1999)

[a] ND, not designed

References

Farrell, D.J., Daggard, G., and Mukkur, T.K. (1999). Nested duplex PCR to detect Bordetella pertussis and Bordetella parapertussis and its application in diagnosis of pertussis in nonmetropolitan Southeast Queensland, Australia. *J Clin Microbiol* 37(3)**,** 606-610.

Hendolin, P.H., Markkanen, A., Ylikoski, J., and Wahlfors, J.J. (1997). Use of multiplex PCR for simultaneous detection of four bacterial species in middle ear effusions. *J Clin Microbiol* 35(11)**,** 2854-2858.

Messmer, T.O., Whitney, C.G., and Fields, B.S. (1997). Use of polymerase chain reaction to identify pneumococcal infection associated with hemorrhage and shock in two previously healthy young children. *Clin Chem* 43(6 Pt 1)**,** 930-935.

Tzanakaki, G., Tsopanomichalou, M., Kesanopoulos, K., Matzourani, R., Sioumala, M., Tabaki, A., et al. (2005). Simultaneous single-tube PCR assay for the detection of Neisseria meningitidis, Haemophilus influenzae type b and Streptococcus pneumoniae. *Clin Microbiol Infect* 11(5)**,** 386-390. doi: 10.1111/j.1469-0691.2005.01109.x.
