# Supplementary material for: Simultaneous Detection of Key Bacterial Pathogens Related to Pneumonia and Meningitis Using Multiplex PCR Coupled With Mass Spectrometry
Source: Front Cell Infect Microbiol. 2018 Apr 5;8:107. doi: 10.3389/fcimb.2018.00107 (PMC5895723; doi:10.3389/fcimb.2018.00107)
Supplement: Supplementary file 5 [file Image2.PDF]

Supplementary Material

Simultaneous Detection of Key Bacterial Pathogens Related to Pneumonia and Meningitis by Using Multiplexed PCR Coupled with Mass Spectrometry

Chi Zhang[1]†, Leshan Xiu[1]†, Yan Xiao[1, 2], Zhengde Xie[3]*, Lili Ren[1, 2]*, Junping Peng[1]*

* these authors are corresponding authors.

† these authors contributed equally to this work.

Correspondence:

Junping Peng, pengjp@hotmail.com

Lili Ren, renliliipb@163.com

Zhengde Xie, zhengdexie@bch.com.cn

Supplementary information

Table S1 Target gene and sequences of amplification primers and extension primers used in the BP-MS method

Table S2 Primers and probes of real-time PCR used in this study

Table S3 Primers of nested PCR used in this study

Fig S1 Evaluation the specificity of the assay of *S. pneumoniae*, *H. influenzae*, *N. meningitidis, K. pneumoniae, A. baumannii,* and *P. aeruginosa*.

Fig S2 Evaluation the specificity of the assay of *S. aureus*, *M. catarrhalis*, *L. pneumophila*, *M. pneumoniae, B. pertussis*, and HBB.

Figure S2

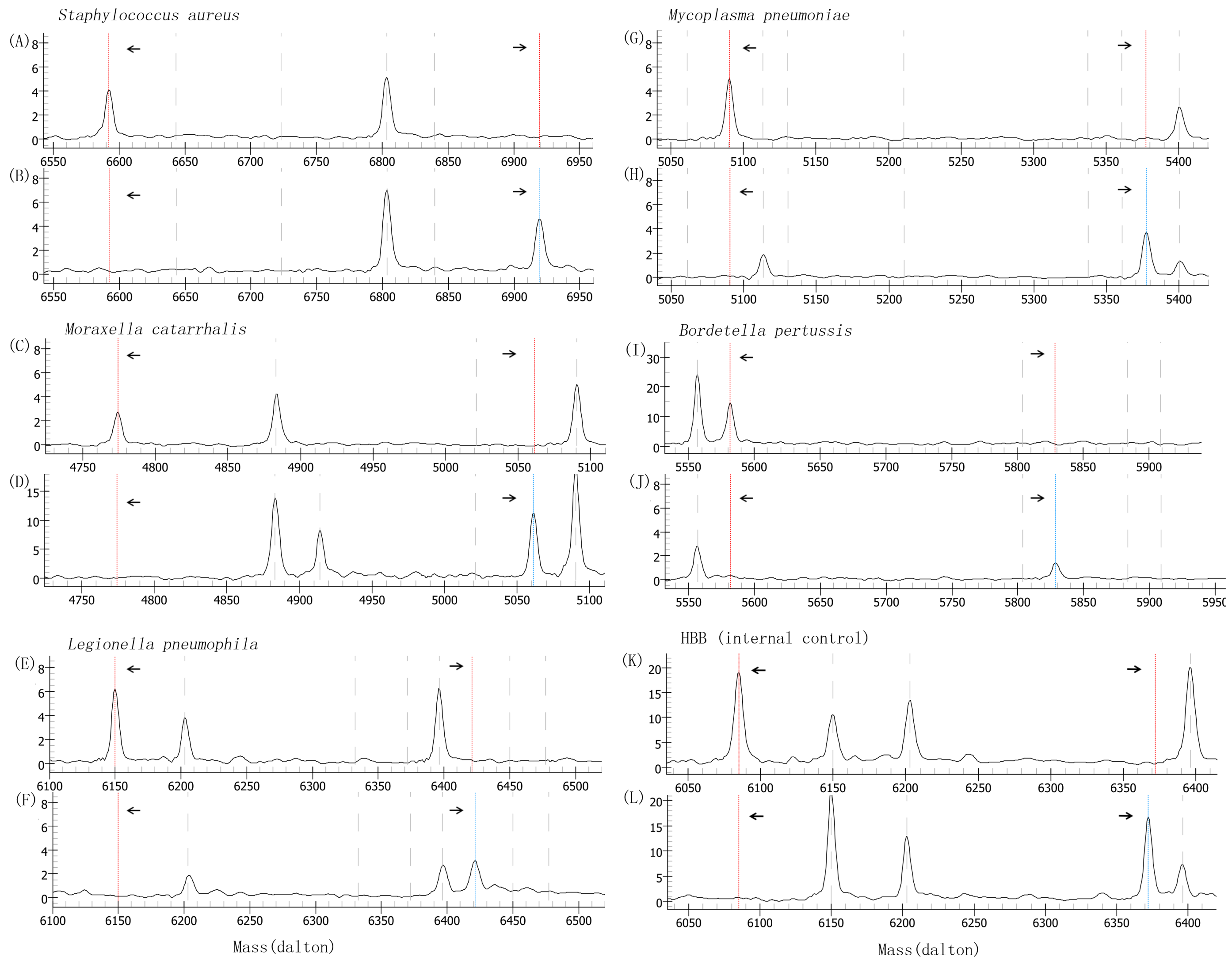

Figure S2 | Mass spectrum of specificity evaluation by using sequencing confirmed clinical samples and isolates. Figure (B), (D), (F), (H), (J) and (L) represent the positive results of *S. aureus*, *M. catarrhalis*, *L. pneumophila*, *M. pneumoniae*, *B. pertussis*, and HBB respectively, while (A), (C), (E), (G), (I) and (K) represent the negative reaction of corresponding assay when a non-target DNA was used. The left arrow indicates the unextended primer and the right arrow indicates the extended primer. The x-axis represents the mass of extension primer and the y-axis represents the intensity.
